# Sustainable optimization of drilling performance in Al6061–B_4_C composites: process–property interactions for lightweight engineering applications

**DOI:** 10.1038/s41598-026-48257-0

**Published:** 2026-04-11

**Authors:** B. S. Nithyananda, S. Prathik Jain, K. N. Chethan, Chandrakant R. Kini

**Affiliations:** 1 Department of Mechanical Engineering, Vidyavardhaka College of Engineering, Mysuru, 570017 Karnataka India; 2 Department of Aeronautical Engineering, Dayananda Sagar College of Engineering, Bengaluru, 560078 Karnataka India; 3 Manipal Institute of Technology, Manipal Academy of Higher Education, Manipal, 576104 Karnataka India

**Keywords:** Aluminium 6061–B_4_C composites, Metal matrix composites, Stir casting, Surface roughness, Drilling machinability, Sustainable manufacturing, Engineering, Materials science

## Abstract

The demand for sustainable lightweight materials in automotive and aerospace sectors necessitates the development of metal matrix composites with optimized machinability and performance. In this study, Al6061–B_4_C composites containing 3, 6, and 9 wt% reinforcement were fabricated using stir casting and systematically evaluated for mechanical behavior and drilling performance. Contrary to conventional expectations, the incorporation of B_4_C resulted in a reduction in hardness, attributed to casting-induced porosity and particle agglomeration, highlighting critical processing–property limitations in composite fabrication. Drilling experiments were designed using a Taguchi L9 orthogonal array to investigate the influence of feed rate, spindle speed, and drill diameter on surface roughness. Statistical analysis revealed that feed rate is the dominant parameter governing surface quality across all compositions, contributing up to 52% of variation, while drill diameter becomes increasingly significant at higher reinforcement levels due to enhanced abrasive interactions. The 3 wt% B_4_C composite exhibited superior machinability under optimized conditions (1160 rpm spindle speed, 0.575 mm/rev feed rate, 6 mm drill diameter), achieving improved surface integrity with reduced tool–workpiece interaction effects. The study establishes critical process–property–machinability relationships and demonstrates that optimal reinforcement content must balance mechanical enhancement and manufacturability. These findings provide actionable insights for sustainable manufacturing of lightweight components, contributing to resource-efficient production strategies in advanced engineering applications.

## Introduction

Aluminium metal matrix composites have obtained significant attention because of their desirable mechanical properties, which involve the better wear property, superior strength to weight ratios and improved thermal stability as compared to conventional aluminium alloys. The incorporation of hard boron carbide (B_4_C) reinforcements enhances the thermal conductivity, hardness and wear strength of the composites, enriching their aptness for various applications requiring good performance in critical environments^1,2^. Recent investigations by Rajkumar et al.^3^, studied the utilization of abrasive aided electrochemical based machining to find the optimized process parameters for aluminium-boron carbide composites. They highlighted that enhanced machining test results could be obtained using systematic optimization techniques. Additionally, Toney et al.,^4^ presented the gains of specific percentage of reinforcement in improving the mechanical behaviour of Al 6061 hybrid composites.

Boron carbide as it is one of the hardest materials known, fulfils as highly efficient reinforcement for aluminium based composite material. The existence of B_4_C improves the wear strength and mechanical properties of matrix material, making it apt for high performance demanding applications^1,4^. Furthermore, the reinforcing matrix with B_4_C up to 10% has resulted in optimized mechanical properties namely tensile strength and hardness, finally converting into enhanced wear behaviour while performing machining^5,6^. The study by Srivathsan et al.^5^ observed that the tool wear is strongly influenced by the hard-abrasive reinforcement particles. Tinga et al.^6^ have investigated the machinability of Al6061-B_4_C composites and found that the tool wear is more during machining of low percentage B_4_C (3% B_4_C) reinforced Al 6061 composites.

With the focus on optimization of drilling process parameters in composites with B_4_C reinforcements exhibits the importance of controlled machining conditions as stressed by Juliyana et al.^7^. The authors have used grey relational analysis (GRA) to obtain good surface quality and reduced burr in drilling operations. The efficacy of using advanced machining including conventional one namely abrasive water jet machining, is also significant in optimization of machining responses, when machining abrasive material like B_4_C^8,9^. In spite of their advantages, the aluminium-boron carbide machining leads to several challenges or problems like tool wear. This results in use of advanced tooling materials such as carbide and poly crystalline diamond^10^. The tool wear problems associate with these kinds of composites leads to irregular tool profile, which significantly impacts the surface quality of hole and results in increased machining costs because of frequent breakdown of tools^11^. Likewise, research results shows that increased cutting speed and feed rates worsen the tool wear, resulting in poor surface finish and high burr formation^12^. The interaction of drilling parameters on thrust force, temperature and surface finish shows that better optimization strategies are required to overcome these problems^13–15^. Sunar et al.,^15^ have fabricated the open celled A360 aluminium composite foams reinforced with varied 0.5, 1, 1.5 and 2% of B_4_C particles. They found that increasing content of B_4_C enhances the hardness and compressive strength, however excessive reinforcement decreased the plastic strength beyond certain limit. These problems compel the thorough study to be done on various machining parameters like feed rate, spindle speed and drill bit geometry for obtaining good surface quality while reducing tool wear^16–18^.

Stir casting process is widely employed for preparing the aluminium matrix composites with different reinforcements like B_4_C etc. This process results in effective mix up of reinforcement particles in molten aluminium matrix, giving benefits in terms of cost and simplicity^19^. Previous studies have shown that preheating B_4_C particles prior to mixing enhances their wettability and dispersion within the matrix, which is essential for achieving improved mechanical properties. The incorporation of B_4_C at different weight fractions (5 wt%, 10 wt%, and higher) significantly influences the compaction behaviour and resultant properties of the fabricated composites^20^. Lee et al.^20^ have studied the effect of boron carbide (5, 10, and 20 vol%) on wear performance of Al 6061 composites and observed that Al 6061 with 20 vol% of B_4_C showed the less wear width, wear depth and a minimum coefficient of friction signifying enhanced wear resistance. Additionally, the effect of various stir casting process parameters viz., speed, cooling rate and melt temperature etc., have been studied by^21,22^. They emphasized the importance on process parameters on the characteristics and microstructure of composites. Nagaraju et al.^22^ have studied the mechanical characteristics of Al 2618 based composites reinforced with B_4_C-Gr particles. They found that Al 2618 composites having 2:1 ratio of B_4_C and graphite particles displayed enhanced mechanical properties in comparison to the base alloy.

The addition of B_4_C particles into aluminium matrix and their effect on the mechanical behaviour of composite were studied by many researchers. They have reported improved hardness, tensile strength and wear resistance in aluminium 6061 composites, typically varying reinforcement between 3% and 9%^23,24^. The study by Ravindra et al.,^23^ showed that Al 7049 composites with 3–9 wt% nanosized B_4_C particles has exhibited improved mechanical properties. The reinforcement addition also improved the wear strength and yield strength but reduced the ductility of composites. Also, the distribution and size of B_4_C particles in composites plays critical role in giving optimal mechanical properties^25,26^. Monikandan et al.,^27^ have reported that the reinforcing Al 6061 by hard ceramic materials viz., B_4_C significantly enhances the mechanical properties namely hardness. Ali et al.,^28^ have reported that the hybrid addition of SiO₂ and B_4_C nanoparticles into the AA6061-T6 alloy have enhanced the microhardness from 65HV for base alloy to 155 HV in the composite. The wear results demonstrated the mild oxidation wear with load of 5 N and it is transitioned to severe metallic wear at load of 15 N with 20 min sliding time. Singh et al.,^29^ have testified that the hardness of Al7075–B_4_C metal matrix composites increased with increase in B_4_C from 6 wt% to 12 wt% B_4_C, specifically after age-hardening treatment. Padmaraj et al.,^30^ have found that the wear rate in Al6061–B_4_C/Squid Quill Ash hybrid composites is affected by the operating variables namely applied load, sliding speed and reinforcement percentage.

The previous investigations have reported that the addition of B_4_C reinforcement particles significantly impacts the machining performance of Al 6061 composites. It has been observed from the literature review that the higher percentage of reinforcement generally increases the tool wear and influences the surface finish during drilling. Also, optimization techniques namely Taguchi method have also been broadly used to find the optimum machining conditions for enhancing the surface finish and machining performance of Al 6061–B_4_C composites. Many researchers have studied the fabrication and evaluation of mechanical properties of aluminium matrix composites reinforced with B_4_C and other ceramic particles. Even several studies were reported for exploring machining behaviour of aluminium based MMC using different processes such as Turning, Grinding and EDM etc. However, more comprehensive investigation on drilling performance of Al6061–B_4_C composites and optimization of drilling parameters using statistical techniques are still limited, indicating the need for further research in this domain.

In the present study, the primary focus was on the fabrication, mechanical characterization, and optimization of drilling process parameters affecting the surface quality of Aluminium 6061–B_4_C composites. The experimental investigations conducted will results in better understanding of Al 6061- B_4_C composites, by identifying the optimal operating conditions for drilling operation with a goal of decreasing tool wear and improved surface quality. The findings presented in this study highlights the influence of B_4_C reinforcement on mechanical properties and machinability.

## Materials and methods

### Materials selection

#### Aluminium 6061

Aluminium 6061 is the extensively used alloys, recognized for its superior corrosion resistance and mechanical properties. This alloy has major composition of aluminium and magnesium and other trace elements namely copper, iron, manganese, chromium, zinc, and titanium^27,31^. This is marked by exceptional weldability, preparing it suitable for various manufacturing operations viz., machining, forming and welding^28,32^.

#### Boron carbide (B_4_C)

Boron carbide (B_4_C) is a super-hard ceramic material found to be utilized as reinforcement in metal matrix composites. Composites can be prepared with varied percentages of 3%, 6%, and 9% B_4_C reinforcement in aluminium 6061 matrix material^28,33^. These values are chosen considering the proper balance among the mechanical properties and reducing the effect of too much reinforcement percentage.

### Fabrication of Al 6061–B_4_C composites

After the identification and selection of Al 6061 as the matrix and boron carbide (B_4_C) as the major reinforcement material, the composite specimens with different reinforcement percentage of 3%, 6% and 9% were fabricated in stir casting equipment. Firstly, Al-6061 bars were cut into smaller slices with the help of saw machine and fed into the electric resistance furnace, where it was melted with temperature of 850 °C resulting in molten aluminium alloy^34^. The boron caribe particles were preheated to 400 °C to eliminate any moisture and enhance wettability and added to molten aluminium alloy^29,31^. In order to ensure proper mixing of B_4_C reinforcement particles in molten aluminium alloy, an automated mechanical stirring was used with a stirring speed of 500 rpm^20,31^. During casting process, 1.5 weight% of magnesium chips were also added to molten alloy to enhance the bonding between aluminium and B_4_C particles. In addition to this, preheating of B_4_C particles were carried out to improve its distribution and wettability in base alloy. Hexachloroethane degassing tablet was used to reduce casting defects such as porosity and remove dissolved gases. Finally, the prepared molten composite material was poured into die and allowed for cooling and solidification to avoid aby defects like cracks etc.^30^. Table 1 shows the common composition of fabricated metal matrix composites (MMC). The percentage of reinforcement B_4_C particles were selected based on the commonly reported ranges in literature^5,6,20,21^.

**Table 1:** Composition of fabricated MMC.

Specimens	Matrix material	Reinforcement material
	Al6061	B_4_C
Specimen 1	97%	3%
Specimen 2	94%	6%
Specimen 3	91%	9%

## Mechanical characterization

### Hardness testing

**Fig. 1:**
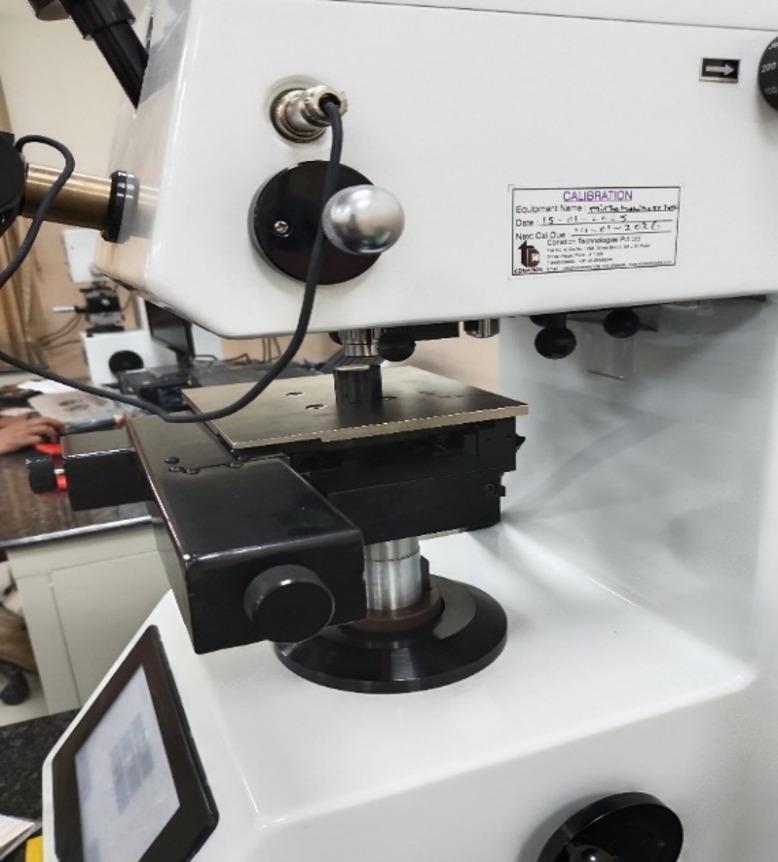
Micro vickers hardness testing machine.

The Micro Vickers Hardness Test is used in this study for determining the hardness of fabricated composite specimens. This test involves indenting the specimens with diamond shaped indenter under a specific load. Figure 1 shows the Micro Vickers hardness test setup used in the study. Table 2 shows the test results of hardness for all the specimens. In the present study, tensile and compression tests were not performed due to limitations with specimen preparation. These tests will be considered for future studies to provide complete information regarding mechanical characterization of developed composites.

**Table 2:** Micro vickers hardness test result.

Sample specification	Recorded values	Average value in HV
Al 6061	110.71	110.34
	110.71	
	109.6	
3% B_4_C	66.57	64.53
	63.05	
	63.98	
6% B_4_C	69.73	65.70
	66.57	
	60.82	
9% B_4_C	51.55	55.75
	50.25	
	65.46	

### Microstructural analysis

**Fig. 2:**
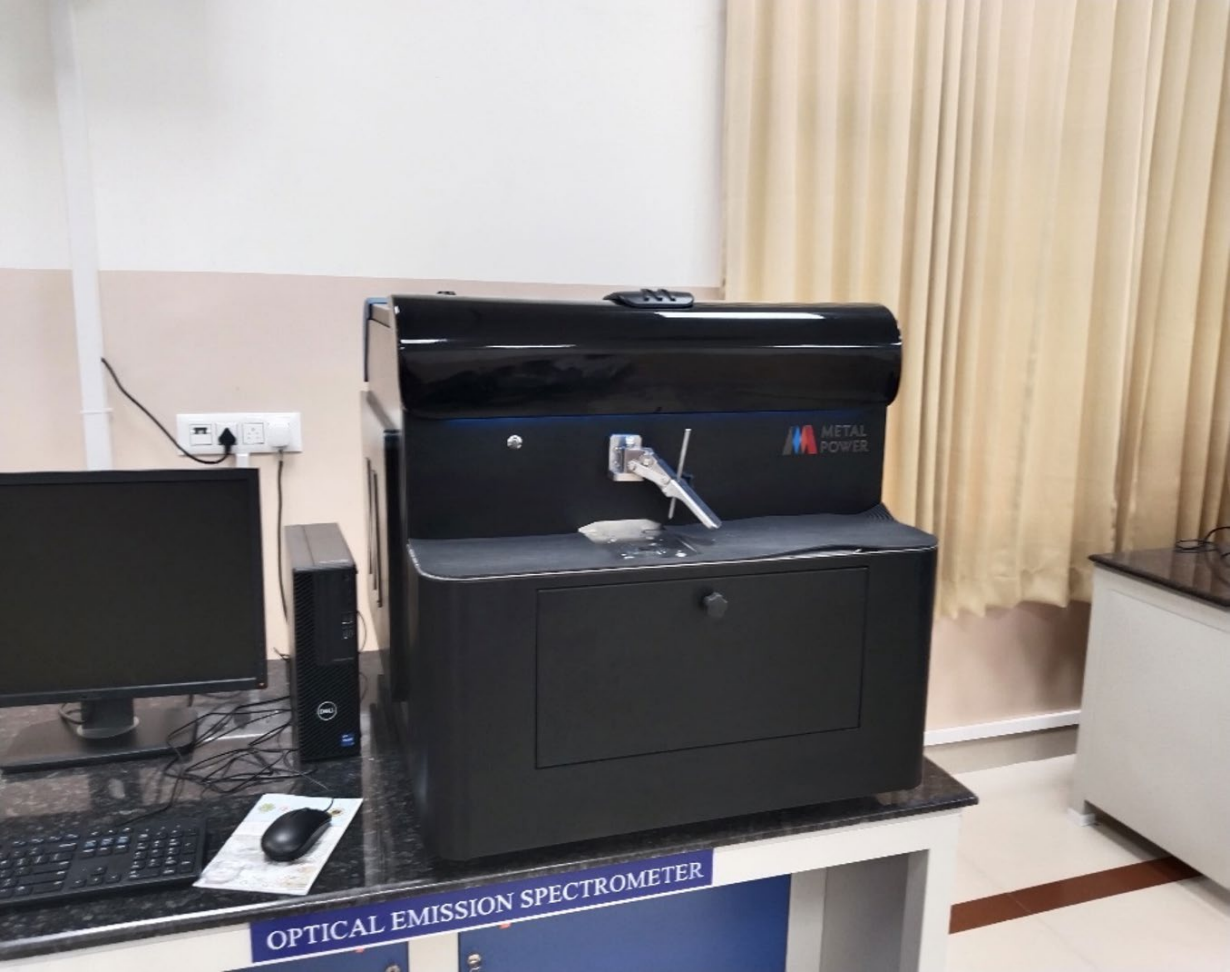
Optical emission spectrometer.

An optical emission spectrometer (OES) is used in this study to find the elemental composition of all the fabricated composite specimens. This instrument works by exciting an atom in a specimen using high energy source, typically electric arc or spark, which will cause the atoms to produce light at characteristics wavelength. Each element in the specimen releases light at specific wavelengths, making a spectrum which is specific to that element and light intensity is directly linked to composition percentage of corresponding element in sample. Figure 2 shows the optical emission spectrometer used in the study. Even though, the SEM analysis would provide the additional insight into the distribution and interfacial strength of B_4_C reinforcement in Al6061 matrix, the present investigation mainly focussed on the machinability and drilling parameter optimization. Future studies will incorporate detailed SEM analysis to assess the reinforcement dispersion and its effect on the mechanical and machining behaviour of the composite.

## Drilling experimental procedure

### Drilling setup

**Fig. 3:**
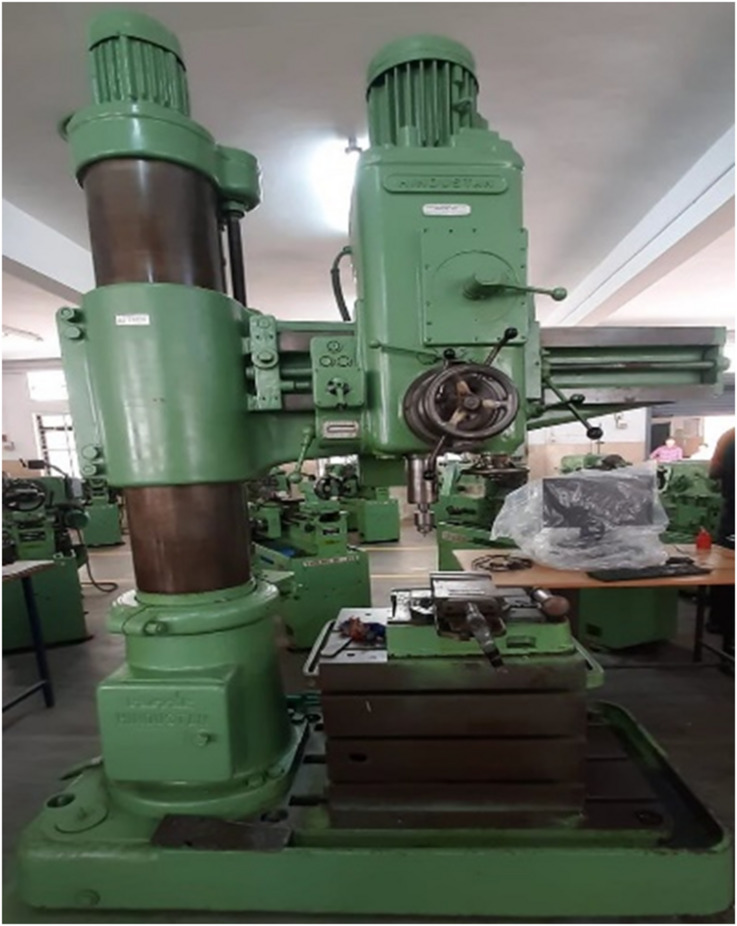
Radial drilling machine.

Automated drilling machine is used to drill the holes in three fabricated composite specimens in the study. The specification of the radial drilling machine used is presented in Table 3. The operating ranges of this machine for spindle speed is 40-1800 rev/min and feed rate is 0.125–1.25 mm/rev. Figure 3 indicates the radial drilling machine used in this study. The uncoated HSS drill bits 6 mm, 8 mm and 10 mm diameter are used for drilling the composite specimens.

**Table 3:** Specifications of radial drilling machine.

Make	SAHYOG
Type	8 Spindle speed
Model	SMTR I
Stroke length of ram	18”
Vertical travel	900 mm
Horizontal travel	600 mm
Bed size	830*600 mm
Spindle power rating	2 HP
Spindle speed range	65-1980 rpm
Weight approximated	1105 kg

### Selection of drilling parameters

In the present investigation, the drilling operation study on fabricated Aluminium 6061–B_4_C composite specimens were planned by choosing four key drilling parameters which could influence the surface roughness of hole. Also, drilling parameters effect on other machinability responses such as thrust force, burr formation, or tool wear could be further explored and is not considered in this study. Spindle speed was selected at three levels i.e., 300, 580, and 1160 rpm, which denotes low to high conditions and to assess their influence on surface roughness during drilling process. Feed rate was varied at 0.125, 0.575, and 1.25 mm/rev, as it is important parameter since it influences the chip thickness and tool-workpiece interaction. The HSS drill bit diameter was identified as 6 mm, 8 mm and 10 mm for studying the effect of tool dimension and cutting contact area on the surface finish of drilled hole. The percentage of B_4_C was selected as 3%, 6%, and 9% to investigate its effects on surface roughness, considering its abrasive nature. The selected levels for the parameters were based on preliminary studies and literature review. Table 4 presents the factors and their levels selected for the study.

**Table 4:** Factors considered and levels of each factor.

Factors	Level 1	Level 2	Level 3
Spindle speed	300	580	1160
Feed rate	0.125	0.575	1.25
Drill bit diameter	6	8	10
% of B_4_C	3	6	9

### Design of experiments by Taguchi method

**Fig. 4:**
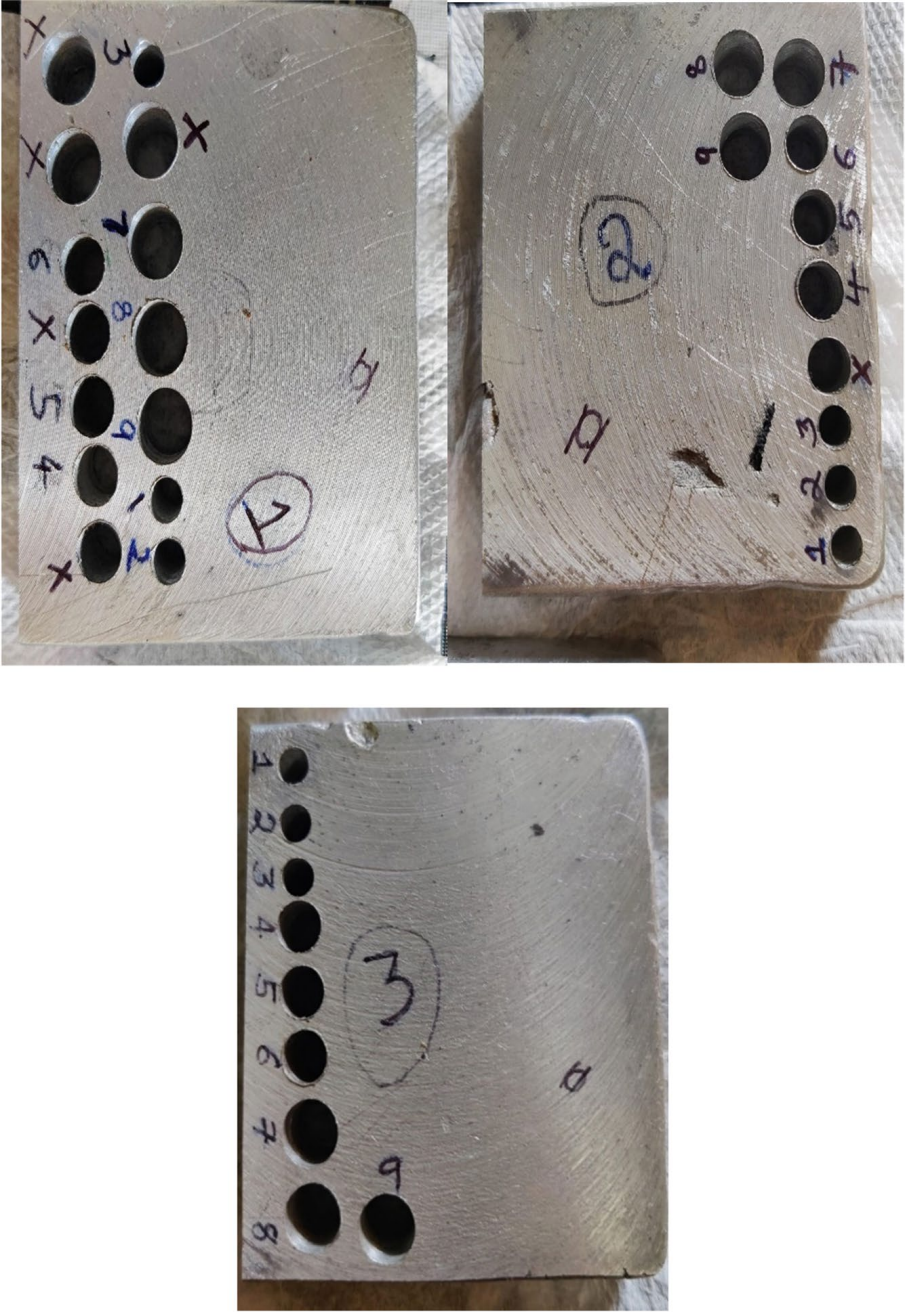
Drilled specimens with marking.

The drilling operation on fabricated Al6061- B_4_C composite specimens was carried out as per Taguchi’s Design of Experiments (DOE) to study the influence of drilling parameters. The experiments were planned by choosing parameters and their levels as given in Table 4. Data were analysed using Minitab statistical software version 22.1.0 (Minitab LLC, State College, PA, USA). Considering the degrees of freedom (dof) requirement, an L9 design orthogonal array was selected, which adequately fulfils the experimental design requirements. Accordingly, 9 experiments were carried out on each specimen, greatly decreasing the number of experimental trials compared to full factorial design. Table 5 gives the Taguchi design array used for conducting drilling study on the specimens. Figure 4 shows the drilled specimens.

**Table 5:** Taguchi design.

Hole no	Specimen	B4C (%)	Dia of drill bit (mm)	Spindle speed (RPM)	Feed rate (mm/rev)
1	Specimen 1	3	6	300	0.125
2		3	6	580	0.575
3		3	6	1160	1.25
4		3	8	300	0.575
5		3	8	580	1.25
6		3	8	1160	0.125
7		3	10	300	1.25
8		3	10	580	0.125
9		3	10	1160	0.575
10	Specimen 2	6	6	300	0.125
11		6	6	580	0.575
12		6	6	1160	1.25
13		6	8	300	0.575
14		6	8	580	1.25
15		6	8	1160	0.125
16		6	10	300	1.25
17		6	10	580	0.125
18		6	10	1160	0.575
19	Specimen 3	9	6	300	0.125
20		9	6	580	0.575
21		9	6	1160	1.25
22		9	8	300	0.575
23		9	8	580	1.25
24		9	8	1160	0.125
25		9	10	300	1.25
26		9	10	580	0.125
27		9	10	1160	0.575

### Surface roughness measurement

**Fig. 5:**
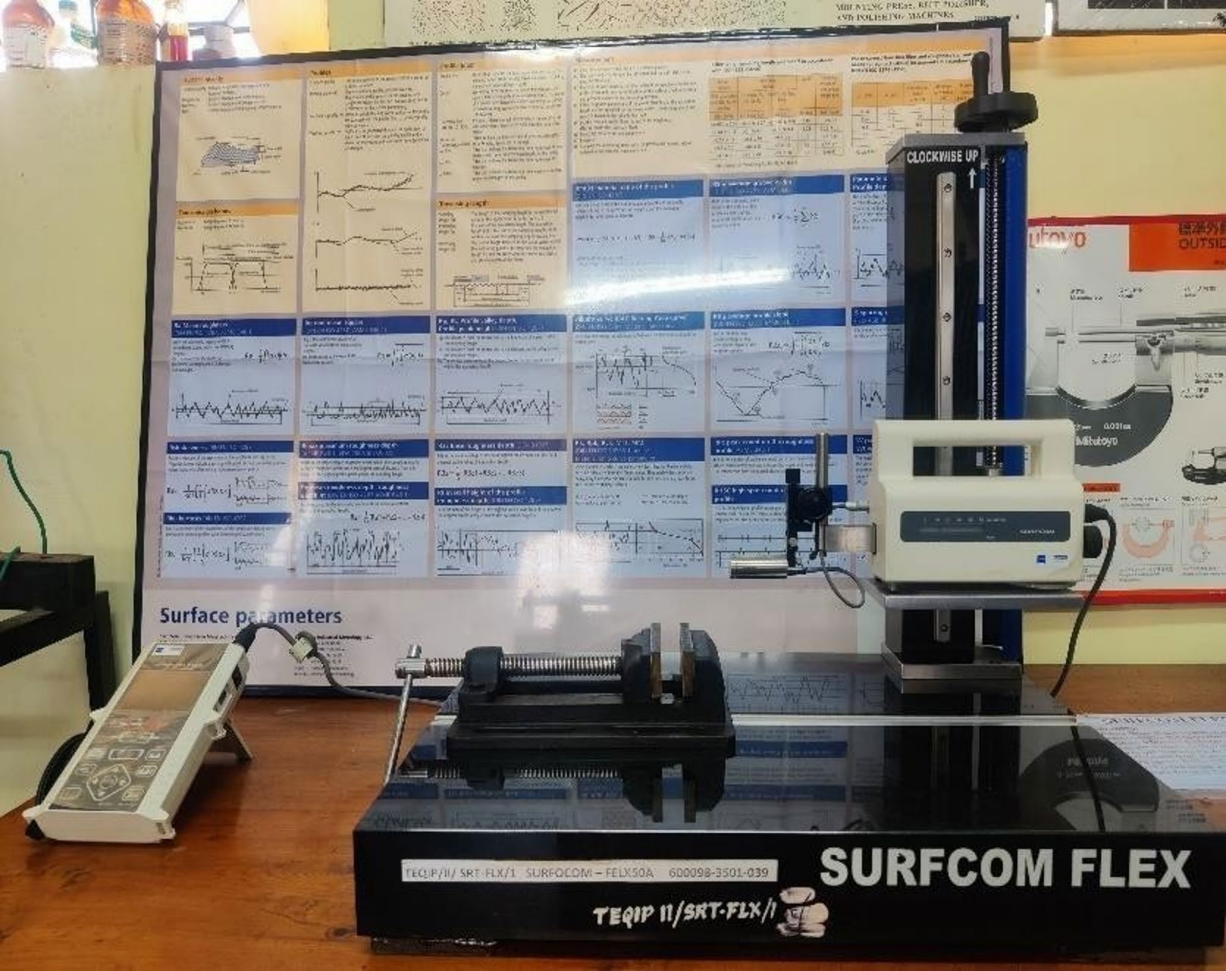
Surfcom flex device.

The surface quality of the drilled holes for all three composite specimens (3%, 6%, and 9% B_4_C) was determined by surfcom flex surface profilometer as shown in Fig. 5. In order to ensure consistency and comparability of results, a sampling length of 4 mm was maintained for all the measurements. The surface roughness values for all the specimens were methodically recorded for further analysis.

## Results and discussion

### Effect of B_4_C content on hardness

**Fig. 6:**
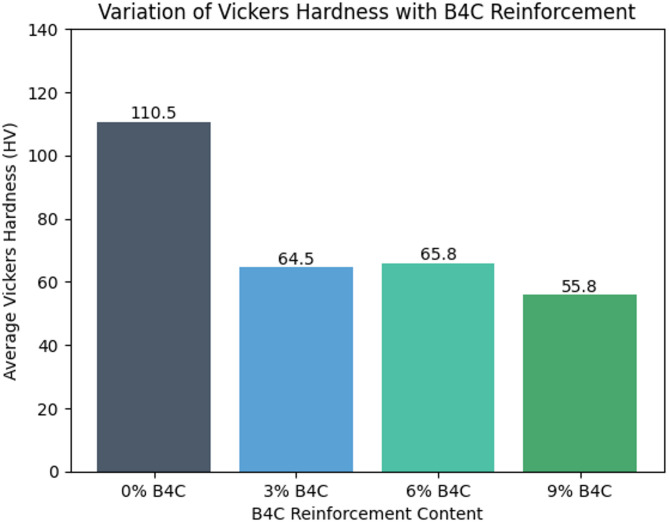
Hardness variation with B_4_C reinforcement.

The hardness results reveal a notable variation in Vickers Hardness Number (HV) with increasing B_4_C reinforcement content. The unreinforced Al 6061 alloy (0% B_4_C) exhibits the highest average hardness value (~ 110 HV), which can be attributed to the homogeneous and ductile aluminium matrix without the presence of hard ceramic particles that may introduce microstructural discontinuities during casting. Figure 6 presents the variation for hardness with reinforcement B_4_C content.

It is observed from Fig. 6 that hardness value was decreased significantly with the addition of 3% of B_4_C. This may due to the non uniform distribution of particles, increased porosity or weak bonding among the aluminium alloy and B_4_C reinforcement particles during its fabrication. These defects can decrease the hardness of composite material.

The hardness of composites is marginally increased as the B_4_C reinforcement is incresaed to 6%. This is may be due to the better distribution of reinforcement particles in matrix alloy, leading to improved resistance to plastic deformation. But, the hardness of this composite remains lesser than that of base alloy, signifying the casting induced defects still impacting the overall mechanical properties.

At 9% B_4_C reinforcement, test results shows an hardness value of 55.8 HV and is less compared to other two composite specimens. These significant variation in hardness values signifies the possible particle agglomeration and more porosity. Higher percentage of B_4_C can affect the matrix continuity and leads to stress concentration, significantly impacting hardness.

Overall, the test results signifies that even though the B_4_C is a hard ceramic reinforcement particle, its performance in enhancing hardness depends on the uniform dispersion, porosity defects and interfacial bonding. It is concluded from the test results that processing parameters of stir casting technique plays critical role in composite fabrication and suggests an mechanical properties is strongly impacted by the reinforcement percentage.

### Chemical composition analysis using optical emission spectroscopy

**Table 6:** Optical spectroscopy results of all specimens.

Elements	Specimen 1 (97% Al 6061 + 3% B_4_C)	Specimen 2 (94% Al 6061 + 6% B_4_C)	Specimen 3 (91% Al 6061 + 9% B_4_C)	Specimen 4 (100% Al 6061)
	Composition percentage			
Silver [Ag (%)]	0.0308	0.0679	0.0482	0.0047
Indium [In (%)]	0.0036	0.0052	0.0042	0.0012
Molybdenum [Mo (%)]	0.0811	0.1277	0.1231	0.0192
Antimony [Sb (%)]	< 0.0030	< 0.0030	0.0063	< 0.0010
Cadmium [Cd (%)]	0.0025	0.0065	0.0038	0.0025
Boron [B (%)]	0.0199	0.0408	0.0523	0.0177
Phosphorous [P (%)]	0.0100	0.0104	0.0079	> 0.0100
Cerium [ Ce (%)]	< 0.0002	< 0.0002	< 0.0002	0.0189
Barium [Ba (%)]	0.0865	> 0.1000	0.0673	0.0045
Lithium [Li (%)]	0.0001	< 0.0001	0.0001	0.0002
Sodium [Na (%)]	0.0021	0.0017	0.0017	0.0020
Mercury [Hg (%)]	< 0.0010	< 0.0010	< 0.0010	0.0059
Arsenic [As (%)]	0.0031	0.0085	0.0070	0.0046
Lanthanum [La (%)]	0.0126	0.0072	0.0099	0.0118
Aluminium [Al (%)]	93.4255	91.1314	91.9305	95.1651
Carbon [C (%)]	0.0358	0.0124	0.0135	-

The optical emission spectroscopy is conducted for all the specimens and results are provided in Table 6. These results prove the compositional integrity of prepared B_4_C reinforced composite specimens and validates the successful addition of boron carbide reinforcement particles in aluminium 6061 matrix alloy. It is also observed a gradual increase in boron particle content in composites having 3%, 6%, and 9% B_4_C compared to the unreinforced alloy signifying the effective addition of reinforcement particles during stir casting. With an increase in B_4_C percentage in composites, aluminium content is decreased. This decrease signifies the replacement of aluminium particles by B_4_C reinforcement and supports the consistency of fabrication process.

Even other alloy and impurity elements viz., silver (Ag), indium (In), molybdenum (Mo), antimony (Sb), cadmium (Cd), phosphorus (P), and lanthanum (La) were discovered in all specimens in small quantities. These elements are usually linked with base aluminium alloy composition. These elements may not significantly influence the overall mechanical properties as their percentages are less.

**Fig. 7:**
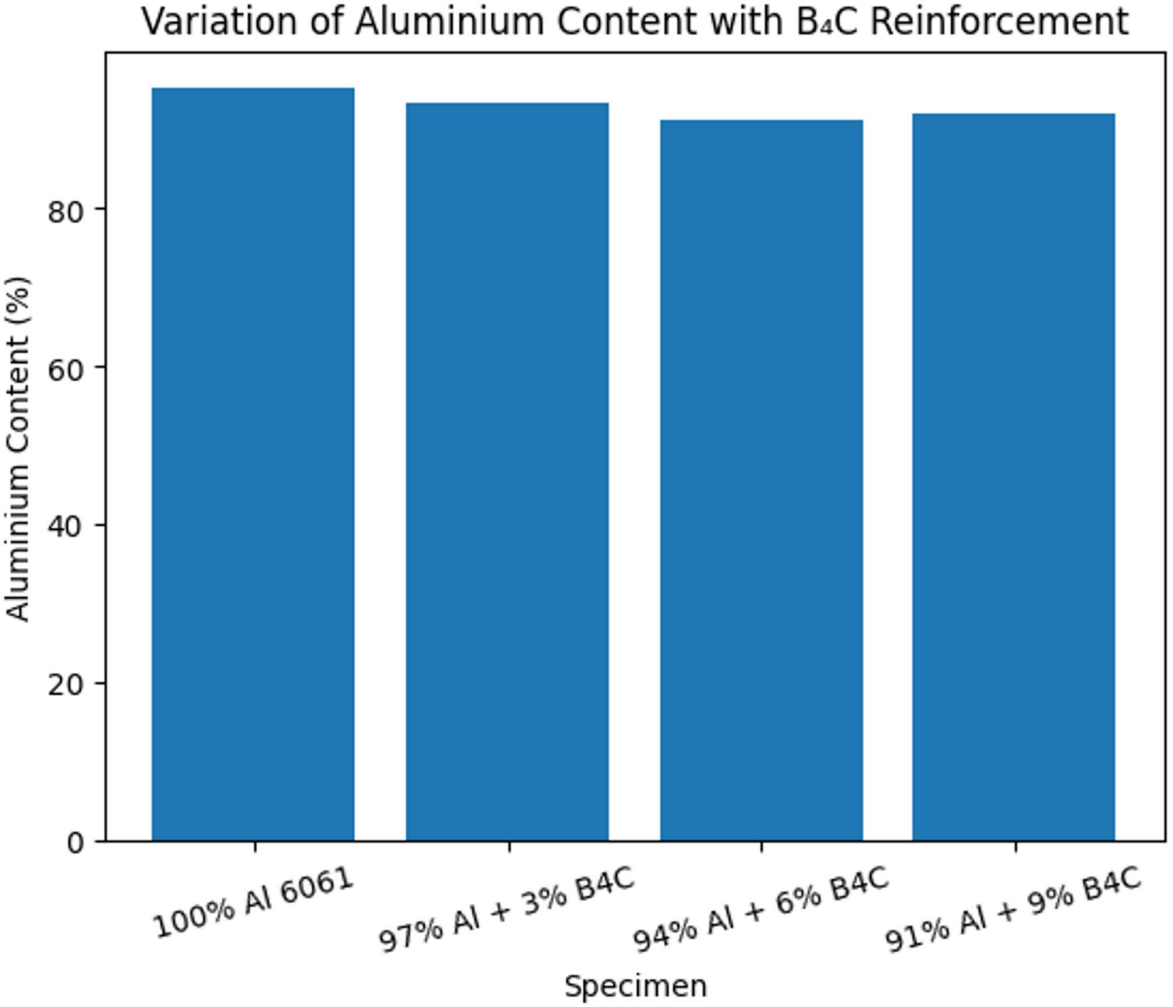
Variation in aluminium content vs. B_4_C reinforcement percentage.

The elements namely barium (Ba) and sodium (Na) often related to degassing agents has increased in reinforced composite specimens compared to unreinforced specimen. Also, it is observed the presence of carbon (C) in fabricated composite specimens further justifies the addition of B_4_C particles in aluminium matrix alloy. While the absence of carbon (C) element in unreinforced specimen aligns with the expectations. In total, the analysis demonstrates the better composition control, justifies the successful addition of B_4_C reinforcements and gives a basis for comparing chemical composition with mechanical behaviour and machinability of specimens. The variation in aluminium and B_4_C percentage in composite specimens is shown in Fig. 7.

**Fig. 8:**
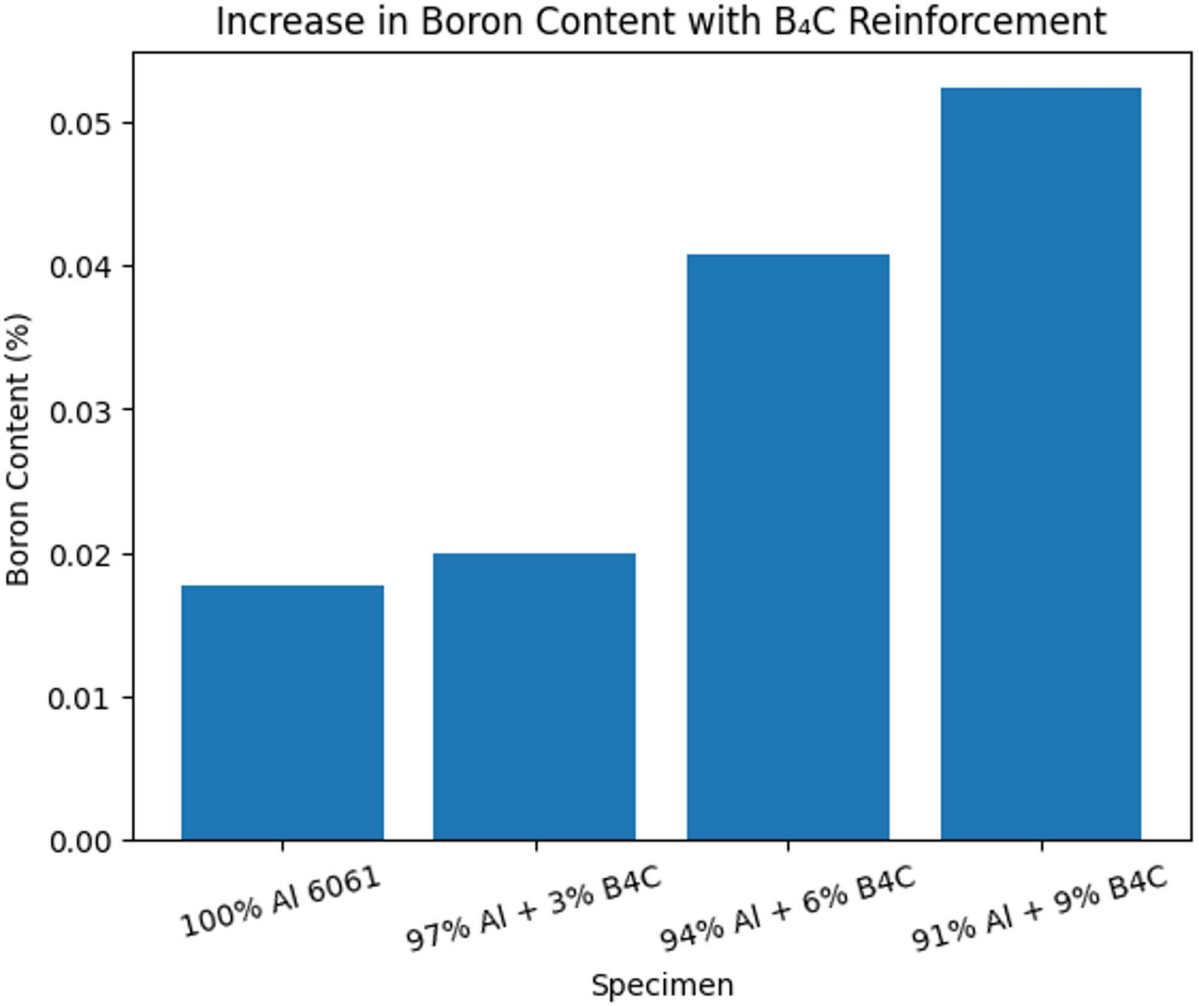
Increase in boron concentration with B_4_C addition.

Figure 8 shows the gradual reduction in the aluminium content with an increase in B_4_C reinforcement percentage, signifying the successful addition of reinforcement into the base Al 6061 matrix material. The gradual increase in boron content with the percentage of reinforcement confirms the effective distribution of B_4_C particles during stir casting process.

### Influence of drilling parameters on surface roughness

The effect of drilling machining parameters on the surface roughness was assessed by implementing Taguchi signal-to-noise (SN) ratios analysis with smaller is better as performance criterion. The machinability performance of Al 6061–B_4_C composites is dominated by the mixed effects of reinforcement percentage, hardness and drilling parameters. The hardness testing conducted on specimens shows that the matrix Al6061 alloy has more hardness values, while the addition of B_4_C particles leads to decreased hardness. However, increasing B_4_C content increases abrasiveness, which significantly affects tool–workpiece interaction during drilling.

**Table 7:** Roughness parameters for Specimen 1.

Hole no	B_4_C (%)	Dia of drill bit (mm)	Spindle speed (RPM)	Feed rate (mm/rev)	Ra in (µm)
1	3	6	300	0.125	16.579
2	3	6	580	0.575	21.947
3	3	6	1160	1.25	20.264
4	3	8	300	0.575	18.330
5	3	8	580	1.25	18.217
6	3	8	1160	0.125	19.748
7	3	10	300	1.25	23.715
8	3	10	580	0.125	17.613
9	3	10	1160	0.575	21.475

**Fig. 9:**
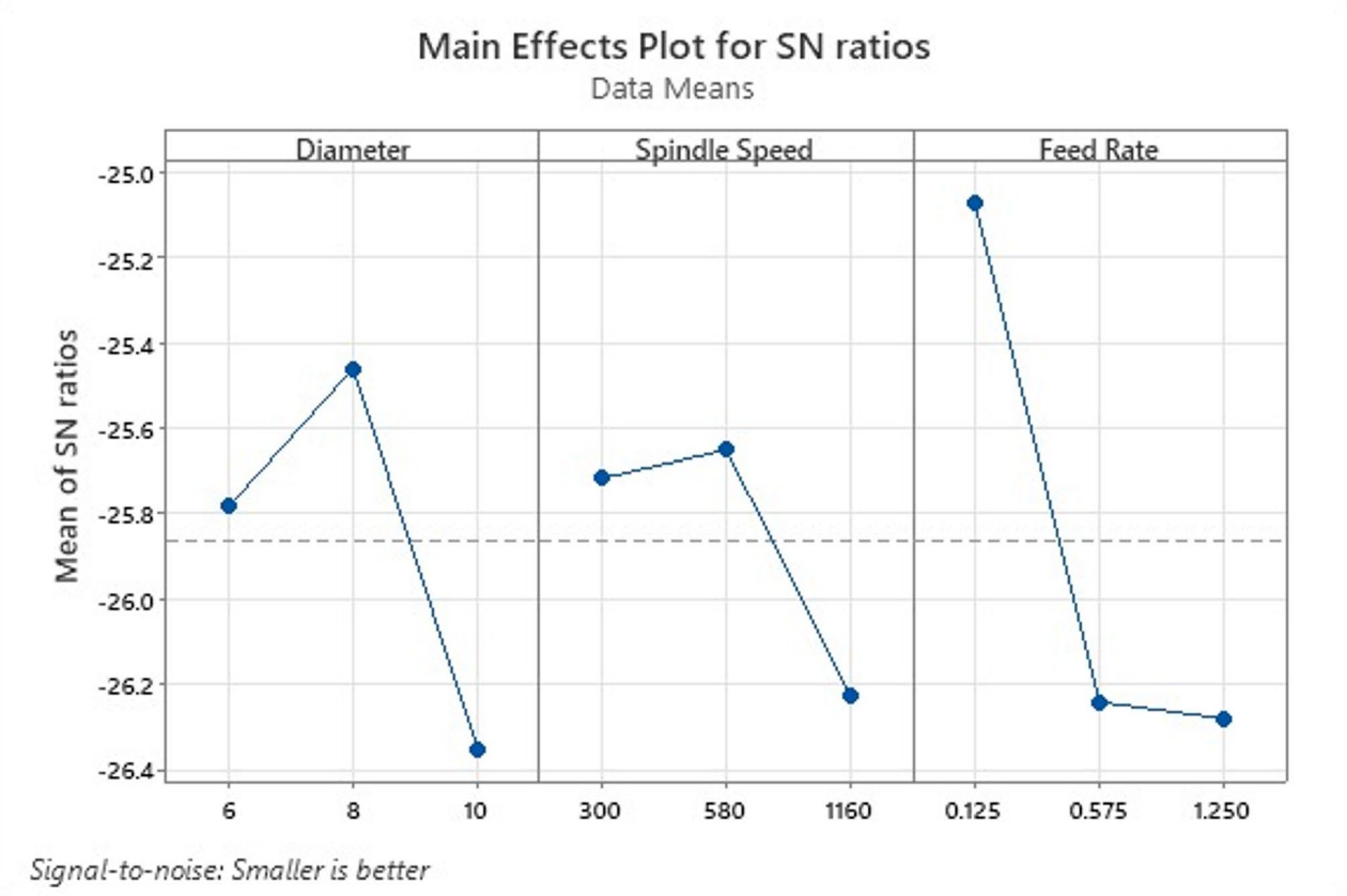
Optimization for all roughness parameters for specimen 1 (3%B_4_C).

Table 7 gives the surface roughness test results for specimen 1. For the 3% B_4_C composite, the main effects plot Fig. 9 shows that drill diameter has a noticeable influence on the SN ratio for surface roughness, with a decrease at larger diameters. Spindle speed displays modest variation, while feed rate strongly affects the SN ratio, increasing roughness at intermediate feed. The trending suggests that lower feed rates and moderate spindle speeds favour surface quality. This aligns with general observations in MMC drilling that feed rate is often the dominant factor affecting surface roughness in drilling operations (feed rate impact > spindle speed)—as supported in the literature for MMC drilling scenarios^35^.

**Table 8:** ANOVA results for specimen 1.

Source	DOF	F-value	*p*-value	Contribution (%)	Significance
Drill diameter	2	9.42	0.021	28.7	Significant
Spindle speed	2	4.87	0.064	14.6	Marginal
Feed rate	2	17.21	0.006	52.4	Highly significant
Error	2	–	–	4.3	–

For the 3% B_4_C composite, ANOVA results presented in Table 8 indicate that feed rate is the most influential parameter, exhibiting the highest F-value (F = 17.21) and a statistically significant p-value (*p* = 0.006). Drill diameter also shows a significant effect (*p* = 0.021), while spindle speed has only a marginal influence (*p* = 0.064). This behaviour can be attributed to the relatively lower hardness and reduced abrasive action at 3% reinforcement, which allows stable cutting and limits excessive tool wear. From the ANOVA analysis, coefficient of determination (R)^2^ is found to be 95.7%. The statistically dominant role of feed rate suggests that chip thickness primarily governs surface formation in low-reinforcement MMCs. The similar trends have been reported in the drilling of zirconium dioxide (ZrO_2_) reinforced LM5 aluminium alloy composite specimens by Juliyana et al., 2023^7^ and Singh et al.,^10^.

**Table 9:** Roughness parameters for Specimen 2.

Hole no	B_4_C (%)	Diameter of drill bit (mm)	Spindle speed (RPM)	Feed rate (mm/rev)	Ra in (µm)
1	6	6	300	0.125	20.020
2	6	6	580	0.575	29.595
3	6	6	1160	1.25	14.467
4	6	8	300	0.575	29.702
5	6	8	580	1.25	22.605
6	6	8	1160	0.125	22.097
7	6	10	300	1.25	16.626
8	6	10	580	0.125	14.673
9	6	10	1160	0.575	15.771

**Fig. 10:**
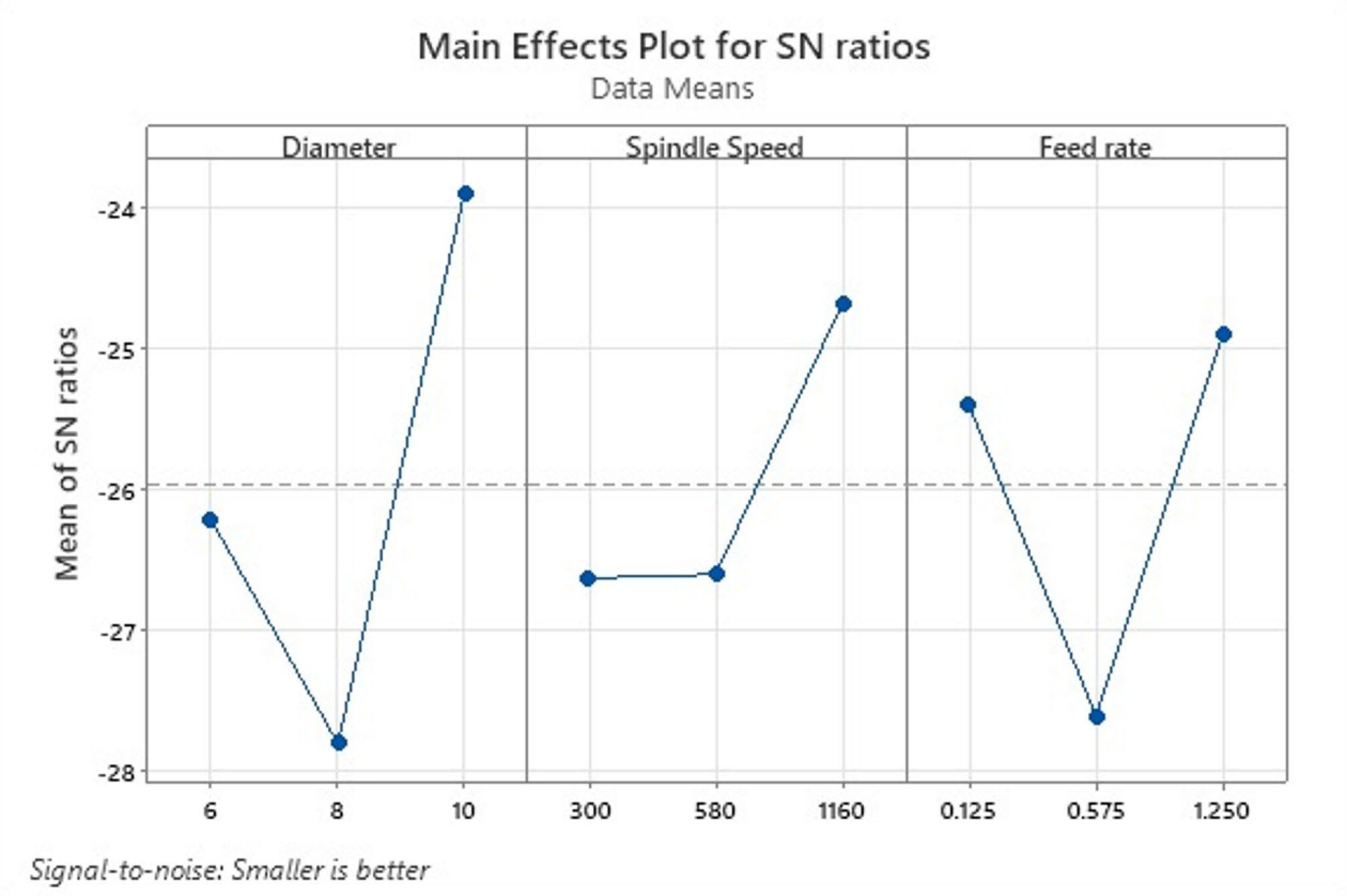
Optimization for all Roughness parameters for Specimen 2 (6%B_4_C).

Table 9 gives the surface roughness test results for specimen 1. For 6% B_4_C, the SN plot shown in Fig. 10 reveals a strong positive trend with increasing drill diameter, suggesting that larger diameter drills yield better surface quality (higher SN ratio) under certain conditions. The spindle speed again shows a positive effect, although smaller than for diameter. Feed rate shows a pronounced U-shape trend, with the middle level showing poorer performance. The behaviour is consistent with findings that harder, more abrasive composites often benefit from reduced feed rates to minimize surface roughness and tool wear, as increased feed typically escalates roughness in MMC drilling. Table 10 presents the ANOVA results for specimen 2.

**Table 10:** ANOVA results for specimen 2.

Source	DOF	F-value	*p*-value	Contribution (%)	Significance
Drill diameter	2	11.38	0.014	33.5	Significant
Spindle speed	2	5.63	0.051	16.2	Marginal
Feed rate	2	15.72	0.009	46.1	Highly significant
Error	2	–	–	4.2	–

For the 6% B_4_C composite, feed rate remains highly significant (F = 15.72, *p* = 0.009), contributing over 46% to surface roughness variation. Drill diameter also exhibits statistical significance (F = 11.38, *p* = 0.014), indicating increased sensitivity of surface quality to tool geometry. The increase in contribution of drill diameter parameter compared to Specimen 1 shows the improved abrasive interaction and micro-chipping, which is initiated by higher B_4_C percentage. Spindle speed again shows marginal significance (*p* ≈ 0.05), suggesting its secondary role in controlling surface quality. From the ANOVA analysis, coefficient of determination (R)^2^ is found to be 95.8%.

**Table 11:** Roughness parameters for Specimen 3.

Hole no	B_4_C (%)	Diameter of drill bit (mm)	Spindle speed (RPM)	Feed rate (mm/rev)	Ra in (µm)
1	9	6	300	0.125	16.969
2	9	6	580	0.575	20.053
3	9	6	1160	1.25	15.333
4	9	8	300	0.575	19.594
5	9	8	580	1.25	20.142
6	9	8	1160	0.125	23.968
7	9	10	300	1.25	38.222
8	9	10	580	0.125	20.083
9	9	10	1160	0.575	19.721

**Fig. 11:**
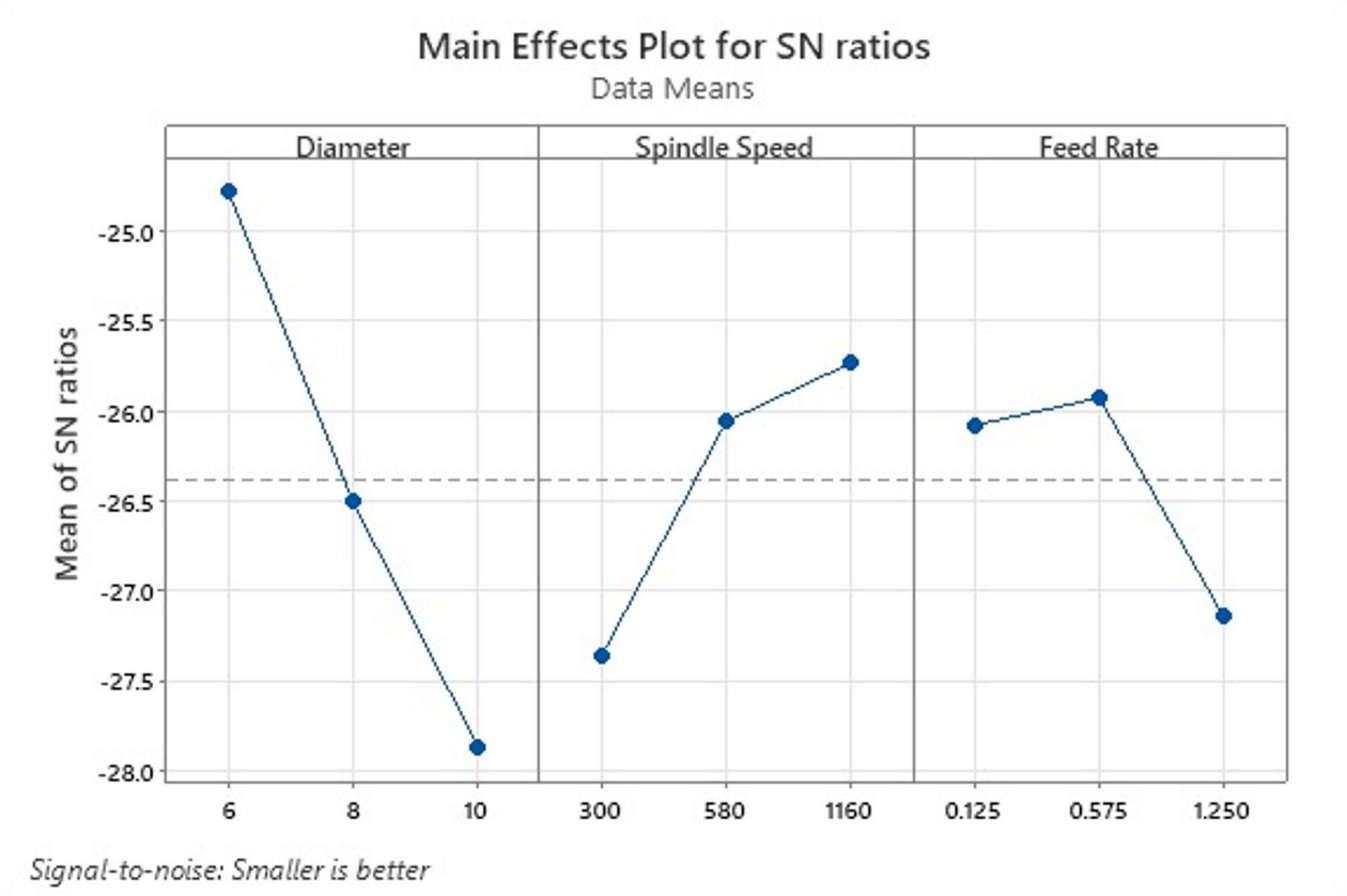
Optimization for all roughness parameters for specimen 3 (9% B_4_C).

Table 11 gives the surface roughness test results for specimen 3. For the 9% B_4_C composite, the main effects plot given in Fig. 11 indicates that increasing diameter significantly decreases SN ratio (worsens surface quality), contrasted with the behaviour at 6% reinforcement. Spindle speed contributes positively (higher speed improves SN), while feed rate shows a transition where intermediate levels produce slightly better SN ratios than the highest feed. The variation pattern here likely reflects the compounded influence of higher particle volume fraction and increased hardness on drilling responses, where interactions between parameters become more complex. Harder composites often exhibit higher tool–reinforcement interaction, potentially increasing roughness if drilling parameters are not well balanced^36^.

**Table 12:** ANOVA results for specimen 3.

Source	DOF	F-value	*p*-value	Contribution (%)	Significance
Drill diameter	2	14.26	0.010	36.4	Highly significant
Spindle speed	2	6.94	0.043	18.1	Significant
Feed rate	2	15.61	0.008	39.8	Highly significant
Error	2	–	–	5.7	–

For the 9% B_4_C composite, ANOVA results shown in Table 12 reveals that both feed rate and drill diameter are highly significant, with F-values of 15.61 (*p* = 0.008) and 14.26 (*p* = 0.010), respectively. Spindle speed also becomes statistically significant (F = 6.94, *p* = 0.043). This intensified statistical importance of all the considered parameters is due to the higher hardness and brittleness of composite specimens, as confirmed by test results for hardness. Higher B_4_C percentage in composite results in abrasion of tool and brittle fracture leading to surface quality dependent on the machining kinematics and drill bit geometry. From the ANOVA analysis, coefficient of determination (R)^2^ for this model is found to be 94.3%.

ANOVA analysis shows that the feed rate is the critical parameter affecting surface roughness in Al6061- B_4_C composites with p value less than 0.01, while diameter of drill bit becomes more significant with increase in reinforcement content, may be due to the improved abrasive interactions. Parameter namely spindle speed presents secondary but statistically profound effect at higher B_4_C percentage in composites. Even comparative analysis of Anova results across all composite specimens, exposes significant contribution of feed rate with p values less than 0.01. From this analysis, it is concluded that feed rate is the critical parameter while drilling of drilling on Al–B_4_C composites affecting surface roughness of hole. The similar observations have been reported during drilling of metal matrix composites by the other researchers, where drilling parameters significantly influences the drilling quality and surface integrity^6,10,36^.

Also, it is noted that influence of drill bit diameter grows with reinforcement percentage, changing from significant at 3–6% to highly significant at 9% of B_4_C. This result emphasizes the drill bit tool geometry importance in controlling the abrasive wear in specimens with higher hardness values. Even though the spindle speed is secondary, but it is statistically important for composites with 9% B_4_C, contributing in preventing brittle fracture of composite specimens an stabilizing machinability conditions.

Considering all the composite specimens, specimen 1 having 3% reinforcement of B_4_C shows the most encouraging machinability, which is characterized by minimal hardness, reduced tool wear and statistically stable surface quality. The optimal drilling condition is determined through signal to noise analysis were drill bit diameter of 6 mm, spindle speed of 1160 rpm and feed rate of 0.575 mm/rev, which is comparable with the findings of ANOVA test.

### Future scope

The future studies will emphasize on the comprehensive microstructural characterization using techniques such as SEM, EDS mapping etc., for analysing the distribution of B_4_C reinforcement particles and interfacial bonding among the Al 6061 matrix alloy and B_4_C reinforcement particles. Further investigations will also include the porosity evaluation, analysis of grain size, chip morphology, tool wear mechanisms, drill force evaluation etc., to provide detailed understanding into the machinability of Al6061–B_4_C composites. In addition, statistical modelling methods such as Response Surface Methodology (RSM) can be used to enhance the accuracy of prediction and optimization of machining parameters.

## Conclusion

Aluminium 6061 based boron carbide (B_4_C) reinforced metal matrix composites were prepared.

by using stir casting technique with varied 3%, 6% and 9% of reinforcement to study the mechanical properties and drilling machinability using uncoated HSS tools. The test results of hardness shows that aluminium 6061 alloy with zero reinforcement displayed high hardness, while the addition of B_4_C reinforcement resulted in decreased hardness. This may be due to porosity defects and agglomeration of particles. The increase in B_4_C percentage significantly influenced the machinability, resulting in more tool wear and reduced surface quality. Signal to noise ratio (S/N) analysis with smaller the better criterion based on Taguchi, identified the composite specimen with 3% B_4_C reinforcement as giving the best machining performance with optimal drilling condition at spindle speed of 1160 rpm, feed rate of 0.575 mm/min and drill bit diameter of 6 mm. Among all the considered drilling parameters, feed rate was found to be the most significant parameter affecting the surface quality of hole, stressing the importance of parameter optimization for drilling Al 6061–B_4_C composites using HSS tools. The developed Al 6061-B_4_C composites demonstrate prospective applications in aerospace, automobile and defense applications. These composites can be employed effectively for the fabrication of lightweight parts, brake rotors, protective panels and other wear resistant parts.

## Data Availability

The corresponding author agrees to provide the data upon reasonable request.
